# Supplementary material for: The gut microbiota directs vitamin A flux to regulate intestinal T cell development
Source: bioRxiv. 2025 Sep 10:2025.09.08.674524. Preprint. [Version 1] doi: 10.1101/2025.09.08.674524 (PMC12440027; doi:10.1101/2025.09.08.674524)
Supplement: Supplement 1 [file NIHPP2025.09.08.674524v1-supplement-1.pdf]

bioRxiv preprint doi: https://doi.org/10.1101/2025.09.08.674524; this version posted September 10, 2025. The copyright holder for this preprint (which was not certified by peer review) is the author/funder, who has granted bioRxiv a license to display the preprint in perpetuity. It is made available under aCC-BY 4.0 International license.

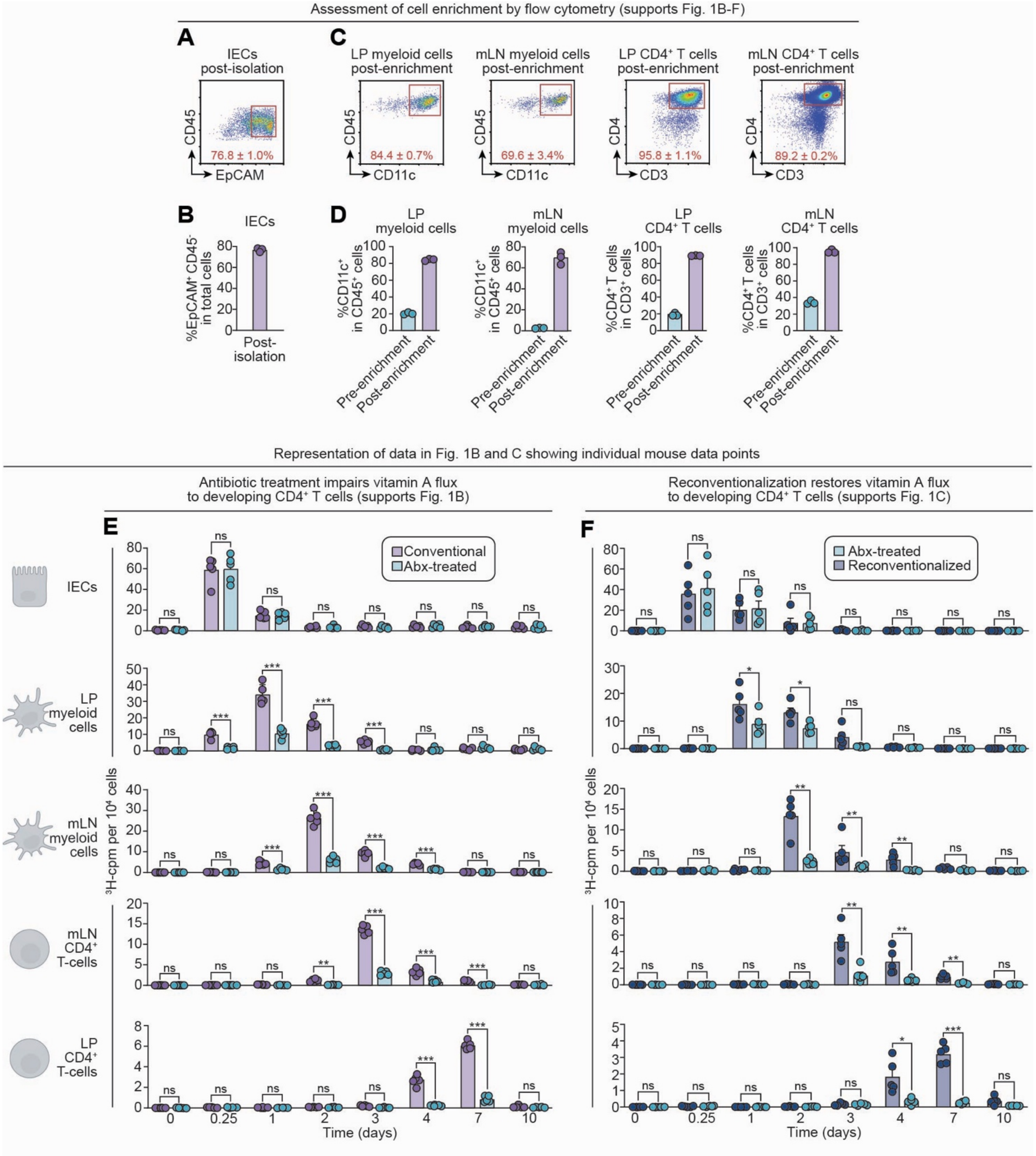

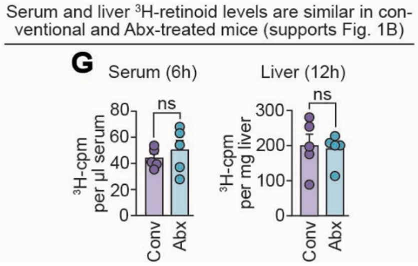

Tissue distribution of absorbed retinoids following oral administration of ³H-retinol to conventional mice (supports Fig. 1B)

H

Cells/tissue	Fraction of total absorbed ^{3}H-retinol (6h)	Fraction of total absorbed ^{3}H-retinol (12h)	Fraction of total absorbed ^{3}H-retinol (24h)
IECs	86.6 ± 9.5%	32.3 ± 1.8%	21.6 ± 1.9%
LP CD11c$^+$ myeloid cells	1.0 ± 0.1%	not detected	1.9 ± 0.2%
Serum	6.6 ± 0.5%	4.7 ± 0.6%	3.0 ± 0.4%
Liver	5.8 ± 0.8%	53.0 ± 5.1%	56.7 ± 2.6%

bioRxiv preprint doi: https://doi.org/10.1101/2025.09.08.674524; this version posted September 10, 2025. The copyright holder for this preprint (which was not certified by peer review) is the author/funder, who has granted bioRxiv a license to display the preprint in perpetuity. It is made available under aCC-BY 4.0 International license.

Figure S1: Purity of enriched immune populations, systemic ^{3}H-retinoid uptake, and full kinetics of retinoid trafficking across the IEC–myeloid–T cell axis (supports Figure 1).

(A) Intestinal epithelial cells (IECs) were isolated by EDTA treatment of mouse small intestinal tissue.[80] Representative flow cytometry plots following EDTA-based isolation are shown. EpCAM marks IECs.

(B) Quantification of IEC purity following EDTA-based isolation.

(C) Lamina propria (LP) and mesenteric lymph node (mLN) CD11c$^+$ myeloid cells and CD4$^+$ T cells were isolated using magnetic bead enrichment. Representative flow cytometry plots of enriched populations are shown.

(D) Quantification of post-enrichment purity.

(E) Measurement of ^{3}H-retinoid flux through IECs, LP CD11c$^+$ myeloid cells, mLN CD11c$^+$ myeloid cells, mLN CD4$^+$ T cells, and LP CD4$^+$ T cells from conventional and antibiotic-treated mice.

(F) Measurement of ^{3}H-retinoid flux in the same cell populations recovered from antibiotic-treated mice and mice that were reconventionalized after antibiotic treatment.

(G) Following gavage of ^{3}H-retinol, serum and liver samples were collected at 6h and 12h, respectively, from conventional (Conv) and antibiotic-treated (Abx) mice. ^{3}H counts per minute (cpm) were normalized to serum volume or liver tissue mass.

(H) Distribution of absorbed ^{3}H-retinol among IECs, LP CD11c$^+$ myeloid cells, serum, and liver at 6, 12, and 24 hours post-gavage. Values are calculated as the percentage of total ^{3}H-retinoid content at the 6-hour time point. Values are presented as means ± SEM

Conv, conventional; Abx, antibiotic-treated; ReConv, reconventionalized; cpm, counts per minute. n=3-5 mice per group. All data are representative of at least two independent experiments. Means ± SEM are plotted. *$p < 0.05$; **$p < 0.01$; ***$p < 0.001$; ns, not significant by two-tailed Student's *t*-test.

bioRxiv preprint doi: https://doi.org/10.1101/2025.09.08.674524; this version posted September 10, 2025. The copyright holder for this preprint (which was not certified by peer review) is the author/funder, who has granted bioRxiv a license to display the preprint in perpetuity. It is made available under aCC-BY 4.0 International license.

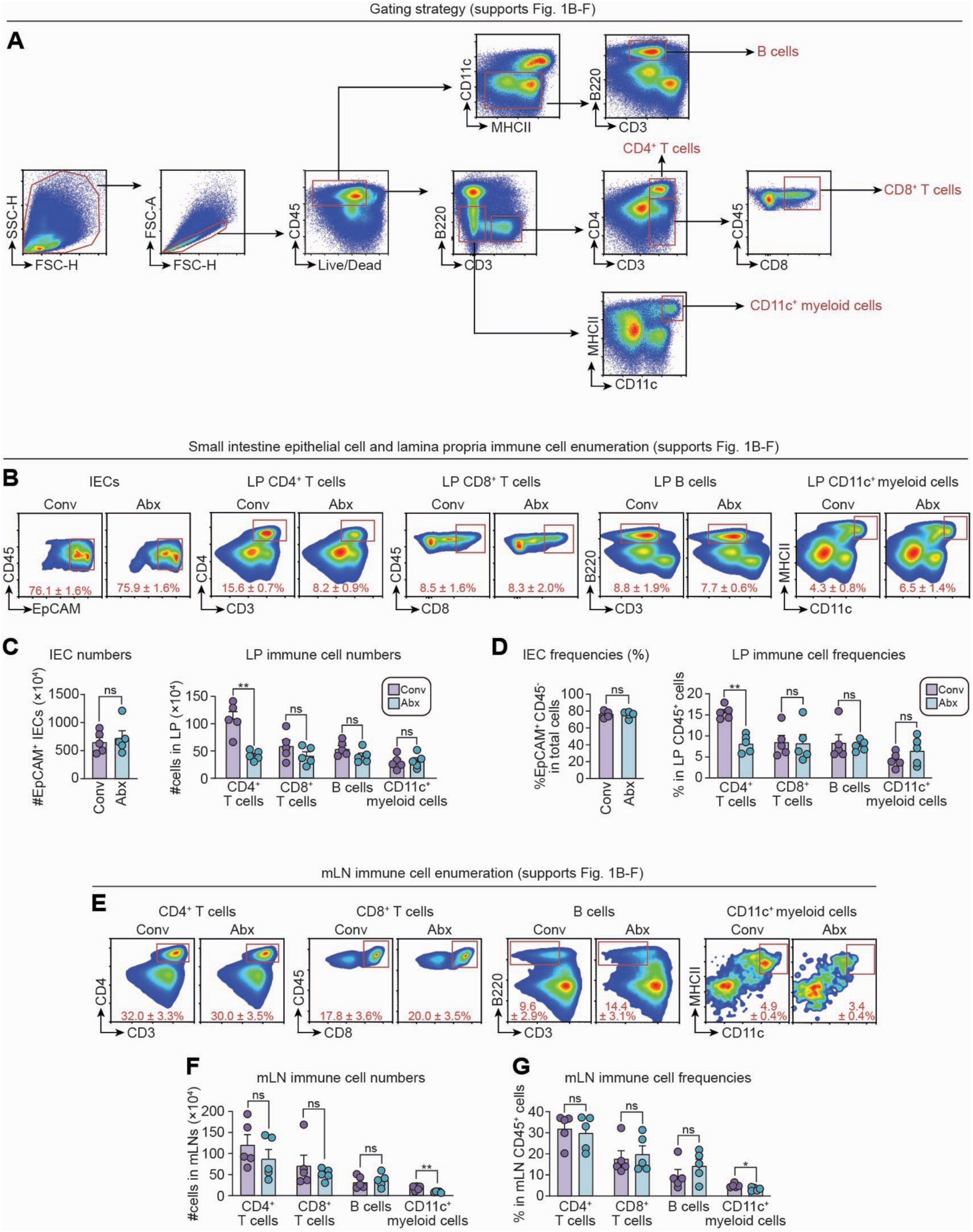

Figure S2. Flow cytometry gating strategy and quantification of epithelial and immune populations in the small intestine and mesenteric lymph nodes of conventional and antibiotic-treated mice (supports Figure 1).

(A) Gating strategy for identifying immune populations in the small intestinal lamina propria and mesenteric lymph nodes. Cell subsets include $CD4^+$ T cells (live $CD45^+B220^-CD3^+CD4^+$), $CD8^+$ T cells (live

bioRxiv preprint doi: https://doi.org/10.1101/2025.09.08.674524; this version posted September 10, 2025. The copyright holder for this preprint (which was not certified by peer review) is the author/funder, who has granted bioRxiv a license to display the preprint in perpetuity. It is made available under aCC-BY 4.0 International license.

$CD45^+B220^-CD3^+CD4^-CD8^+$), B cells (live $CD45^+B220^+CD3^-CD11c^-$), and $CD11c^+$ myeloid cells (live $CD45^+CD3^-B220^-CD11c^+MHCII^+$). Representative flow cytometry plots are shown.

(B) Flow cytometry analysis of IECs (live $EpCAM^+$ $CD45^-$) and lamina propria immune cells recovered from the small intestines of conventional and antibiotic-treated mice. Representative flow cytometry plots are shown.

(C) Absolute numbers of IECs and LP immune cells from conventional and antibiotic-treated mice.

(D) Relative frequencies of each cell population. % IECs is calculated as a percentage of the total cell population, and % LP immune cells is calculated as a percentage of total $CD45^+$ cells.

(E) Flow cytometry analysis of $CD4^+$ T cells, $CD8^+$ T cells, B cells, and $CD11c^+$ myeloid cells recovered from mesenteric lymph nodes of conventional and antibiotic-treated mice. Representative flow cytometry plots are shown.

(F) Absolute numbers of each mLN immune cell population from conventional and antibiotic-treated mice.

(G) Relative frequencies of each mLN immune population (as a percentage of $CD45^+$ cells) in conventional and antibiotic-treated mice.

Conv, conventional; Abx, antibiotic-treated; IEC, intestinal epithelial cells; LP, lamina propria; mLN, mesenteric lymph nodes. n=5 mice per group. All data are representative of at least two independent experiments. Means ± SEM are plotted. *$p < 0.05$; **$p < 0.01$; ns, not significant by two-tailed Student's *t*-test.

bioRxiv preprint doi: https://doi.org/10.1101/2025.09.08.674524; this version posted September 10, 2025. The copyright holder for this preprint (which was not certified by peer review) is the author/funder, who has granted bioRxiv a license to display the preprint in perpetuity. It is made available under aCC-BY 4.0 International license.

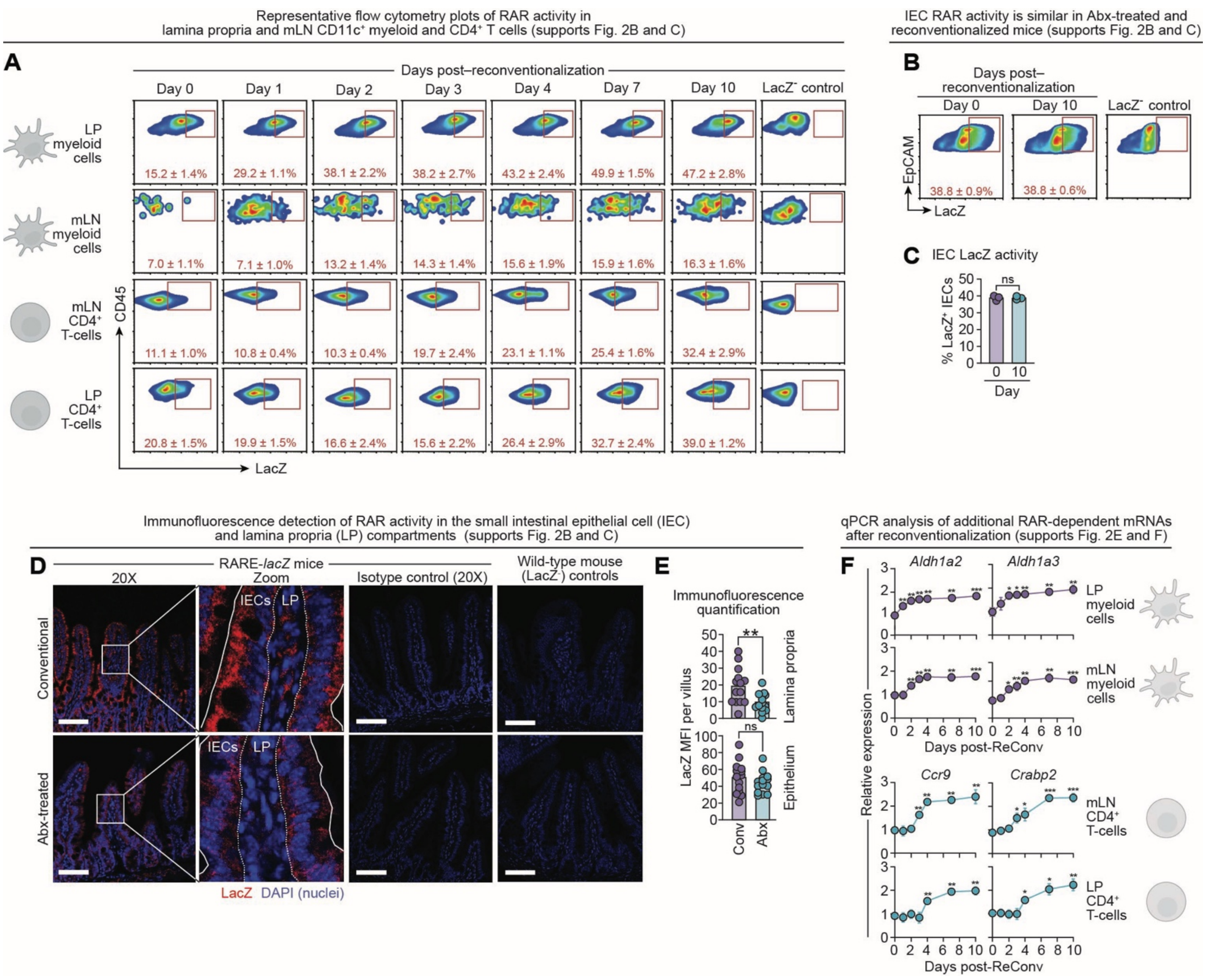

Figure S3: Time course of RAR activity and retinoid metabolism gene expression in CD11c⁺ myeloid cells and CD4⁺ T cells following microbiota reconstitution (supports Figure 2).

- **(A)** Representative flow cytometry plots showing LacZ⁺ (RAR⁺) cell frequencies at the indicated time points post-reconventionalization. Populations were gated as previously described: CD11c⁺ myeloid cells (live CD45⁺CD3⁻B220⁻CD11c⁺MHCII⁺) and CD4⁺ T cells (live CD45⁺B220⁻CD3⁺CD4⁺). LacZ activity was then plotted relative to CD45 expression.
- **(B)** Flow cytometry identification of RAR⁺ intestinal epithelial cells (IECs) at day 0 and 10 post-reconventionalization. IECs were gated as live EpCAM⁺CD45⁻. LacZ activity was then plotted relative to CD45 expression. Representative flow cytometry plots are shown.
- **(C)** Frequency of RAR⁺ IECs at day 0 and day 10 post-reconventionalization. n=3 mice per group per time point.
- **(D)** Immunofluorescence detection of LacZ in the small intestine of conventional and antibiotic-treated RARE-*lacZ* mice. Nuclei stained with DAPI. Scale bars: 100 µm (20X). Isotype and LacZ⁻ controls are shown.
- **(E)** Quantification of LacZ signal (MFI: mean fluorescence intensity) in the lamina propria and epithelial compartments. MFI of LacZ detection was determined for each villus across three fields per mouse. n=5 mice per group.
- **(F)** Time-course of RAR-dependent gene expression as measured by qPCR in LP CD11c⁺ myeloid cells, mLN CD11c⁺ myeloid cells, mLN CD4⁺ T cells, and LP CD4⁺ T cells.

RAR, retinoic acid receptor; IEC, intestinal epithelial cells; Conv, conventional; Abx, antibiotic-treated; ReConv, reconventionalized; LP, lamina propria; mLN, mesenteric lymph node. n=3-5 mice per group. Means ± SEM are plotted. *$p < 0.05$; **$p < 0.01$; ***$p < 0.001$; ns, not significant by two-tailed Student's *t* test.

bioRxiv preprint doi: https://doi.org/10.1101/2025.09.08.674524; this version posted September 10, 2025. The copyright holder for this preprint (which was not certified by peer review) is the author/funder, who has granted bioRxiv a license to display the preprint in perpetuity. It is made available under aCC-BY 4.0 International license.

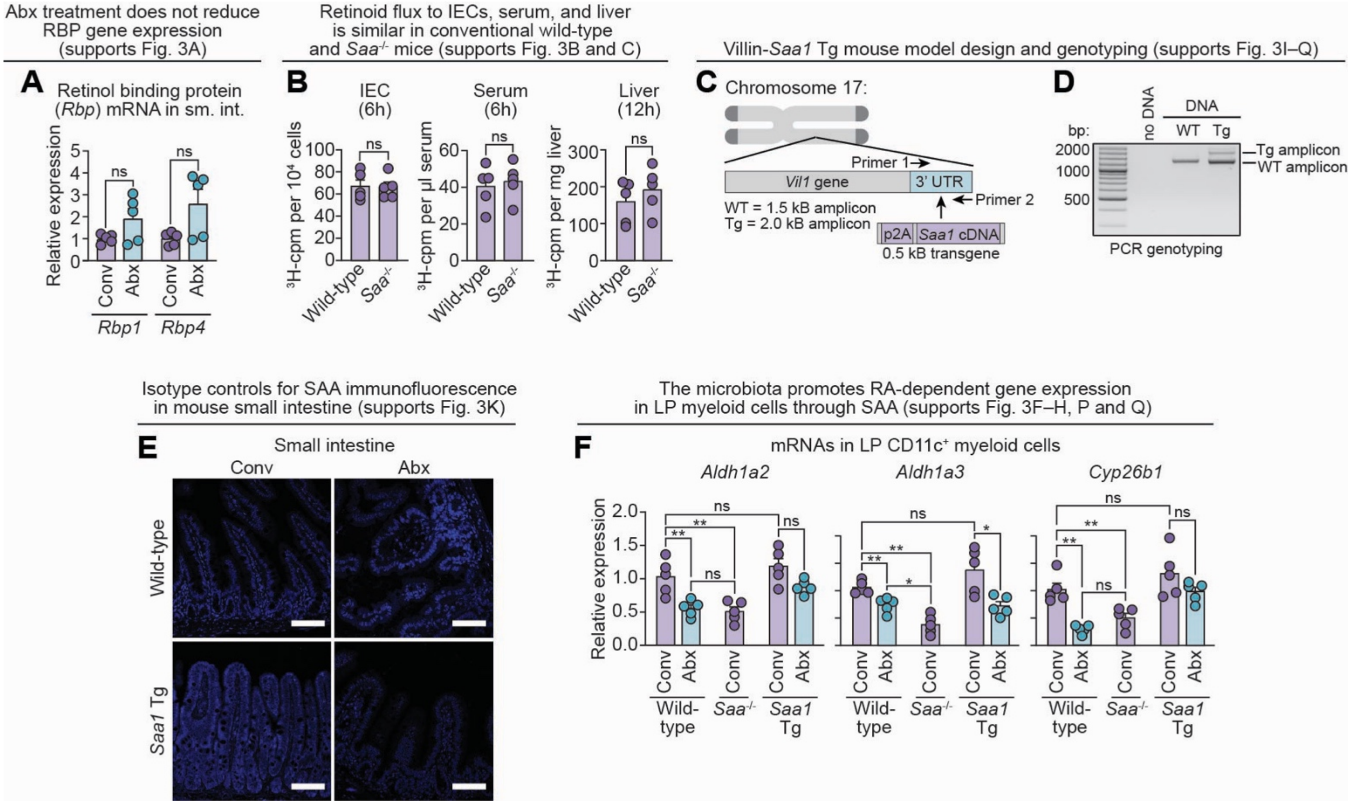

Figure S4: SAA-dependent regulation of intestinal retinol transport, systemic retinoid uptake, and myeloid cell retinoid metabolism (supports Figure 3).

(A) qPCR analysis of *Rbp1* and *Rbp4* mRNAs in the small intestine of conventional and antibiotic-treated wild-type mice. *Rbp* genes encode a family of retinol-binding proteins (RBPs) that are distinct from the serum amyloid A family and that circulate in the bloodstream and within tissues.[43]

(B) ^{3}H-retinoid uptake (normalized cpm per 10^4 cells) in intestinal epithelial cells (IECs) 6 hours post-gavage. ^{3}H-retinoid levels (normalized to volume) in serum at 6 hours post-gavage. ^{3}H-retinoid levels (normalized to tissue mass) in the liver at 12 hours post-gavage.

(C) Schematic of the Villin-*Saa1* transgene (*Saa1* Tg) construct. The *Saa1* transgene is inserted with a preceding p2A site into the 3′ UTR of the *Vil1* (Villin) gene to enable bicistronic transcription of *Vil1* and *Saa1* in intestinal epithelial cells.[45]

(D) PCR genotyping of wild-type and *Saa1* Tg mice.

(E) Isotype controls for SAA immunofluorescence in ileal sections from conventional and antibiotic-treated wild-type and *Saa1* Tg mice. Scale bars = 100 µm.

(F) qPCR analysis of RA-dependent genes in lamina propria CD11c$^+$ myeloid cells from conventional and antibiotic-treated wild-type, conventional *Saa*$^{-/-}$, and conventional and antibiotic-treated *Saa1* Tg mice.

RBP, retinol-binding protein; IEC, intestinal epithelial cells; Tg, transgenic; Conv, conventional; Abx, antibiotic-treated; RA, retinoic acid; LP, lamina propria. n=3-5 mice per group. Means ± SEM are plotted. $^{**}p < 0.01$; ns, not significant by two-tailed Student's *t* test.

bioRxiv preprint doi: https://doi.org/10.1101/2025.09.08.674524; this version posted September 10, 2025. The copyright holder for this preprint (which was not certified by peer review) is the author/funder, who has granted bioRxiv a license to display the preprint in perpetuity. It is made available under aCC-BY 4.0 International license.

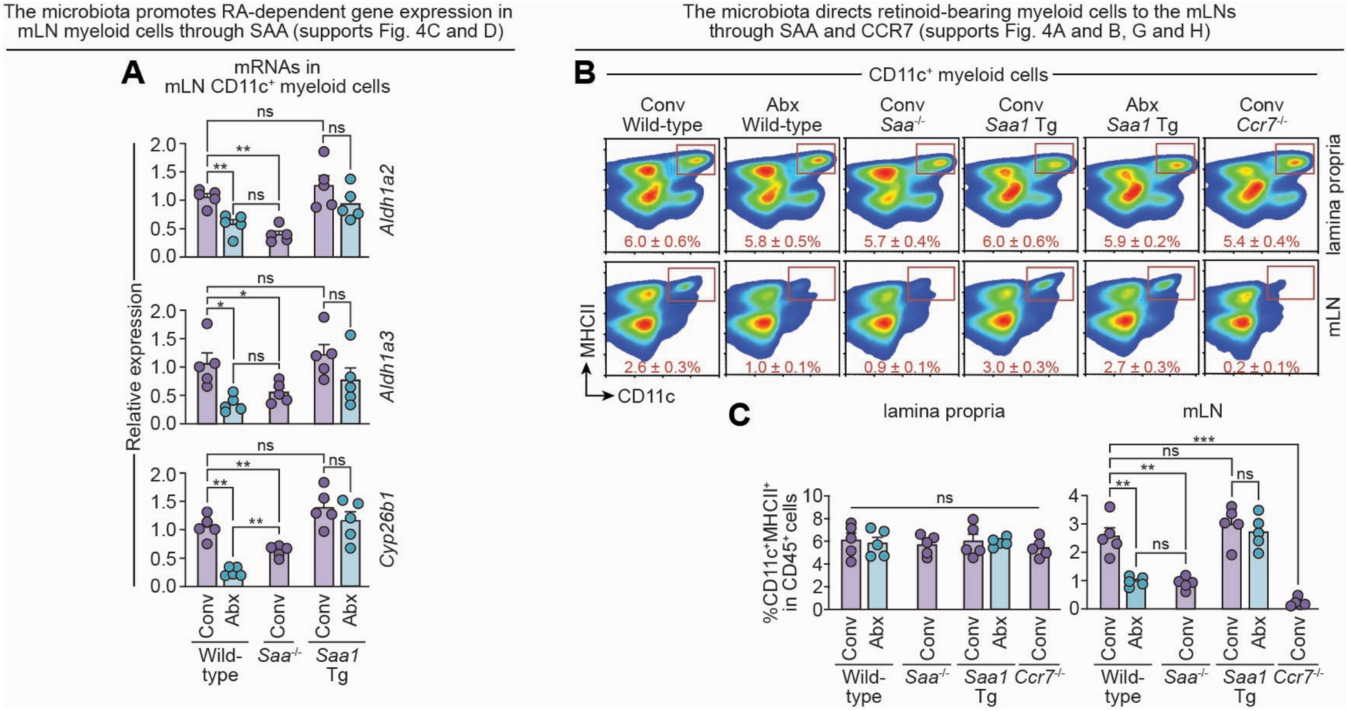

Figure S5: The microbiota directs retinoid-bearing myeloid cells to the mLNs (supports Figure 4).

(A) qPCR quantification of RA-dependent mRNAs in magnetically enriched mLN CD11c+ myeloid cells from conventional and antibiotic-treated wild-type mice, conventional *Saa*$^{-/-}$ mice, and conventional and antibiotic-treated *Saa1* Tg mice.

(B) CD11c+ myeloid cell enumeration by flow cytometry in the lamina propria and mesenteric lymph nodes of conventional and antibiotic-treated wild-type mice, conventional *Saa*$^{-/-}$ mice, conventional and antibiotic-treated *Saa1* Tg mice, and conventional *Ccr7*$^{-/-}$ mice. Representative flow cytometry plots are shown.

(C) Quantification of flow cytometry data in (B).

RA, retinoic acid; mLN, mesenteric lymph nodes; Conv, conventional; Abx, antibiotic-treated. n=4-5 mice per group. All data are representative of at least two independent experiments. Means ± SEM are plotted. *$p < 0.05$; **$p < 0.01$; ***$p < 0.001$; ns, not significant by two-tailed Student's *t*-test

bioRxiv preprint doi: https://doi.org/10.1101/2025.09.08.674524; this version posted September 10, 2025. The copyright holder for this preprint (which was not certified by peer review) is the author/funder, who has granted bioRxiv a license to display the preprint in perpetuity. It is made available under aCC-BY 4.0 International license.

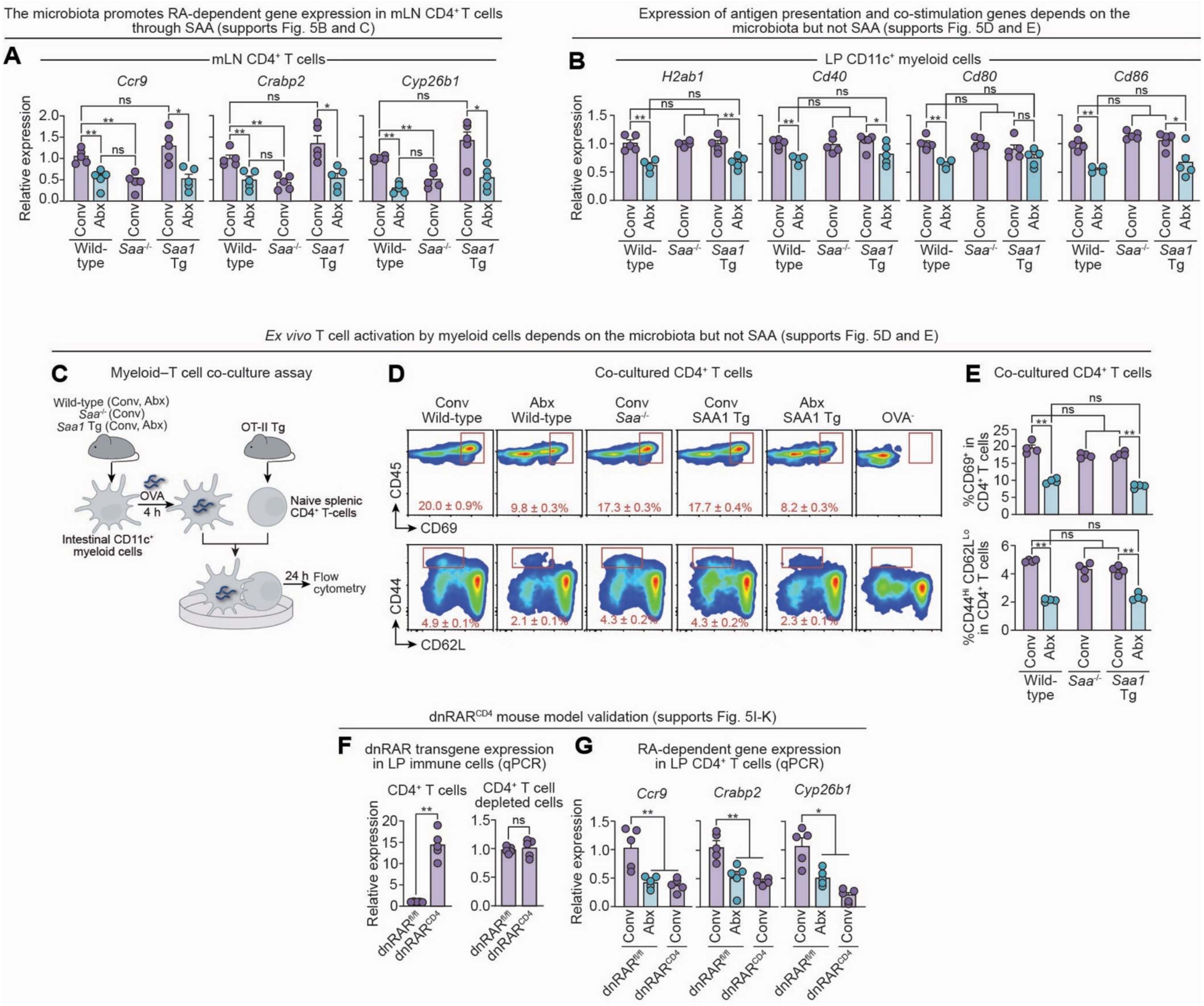

Figure S6: Role of the microbiota and SAA in myeloid cell antigen presentation, T cell activation, and gene expression (supports Figure 5).

(A) qPCR quantification of RA-dependent mRNAs in magnetically enriched mLN CD4$^+$ T cells from conventional and antibiotic-treated wild-type mice, conventional *Saa*$^{-/-}$ mice, and conventional and antibiotic-treated *Saa1* Tg mice.

(B) qPCR quantification of mRNAs associated with antigen presentation and co-stimulatory molecules in LP CD11c$^+$ myeloid cells isolated from the indicated mouse groups.

(C) Strategy for myeloid–T cell co-culture assay. LP CD11c$^+$ myeloid cells were isolated from conventional and antibiotic-treated wild-type mice, conventional *Saa*$^{-/-}$ mice, and conventional and antibiotic-treated *Saa1* Tg mice, pulsed with $OVA_{323\text{-}339}$ peptide, and co-cultured with naïve splenic OT-II CD4$^+$ T cells. T cells were then analyzed by flow cytometry.

(D) CD4$^+$ T cells co-cultured with myeloid cells as described in (C) were analyzed by flow cytometry. CD69 (top) and CD44 and CD62L (bottom) were used as markers of activation.

(E) Quantification of the flow cytometry data in (D).

(F) qPCR quantification of dnRAR transgene expression in CD4$^+$ T cells recovered from the small intestine lamina propria of dnRAR$^{fl/fl}$ and dnRAR^{CD4} mice (left). The same cell population depleted of CD4$^+$ T cells by magnetic separation was also analyzed (right).

bioRxiv preprint doi: https://doi.org/10.1101/2025.09.08.674524; this version posted September 10, 2025. The copyright holder for this preprint (which was not certified by peer review) is the author/funder, who has granted bioRxiv a license to display the preprint in perpetuity. It is made available under aCC-BY 4.0 International license.

(G) qPCR quantification of RA-dependent mRNAs in small intestinal $CD4^+$ T cells from conventional and antibiotic-treated $dnRAR^{fl/fl}$ and $dnRAR^{CD4}$ mice.

RA, retinoic acid; mLN, mesenteric lymph nodes; LP, lamina propria; Conv, conventional; Abx, antibiotic-treated; Tg, transgenic; $OVA_{323-339}$, Ovalbumin peptide 323-339. n=4-5 mice per group. All data are representative of at least two independent experiments. Means ± SEM are plotted. *$p < 0.05$; **$p < 0.01$; ns, not significant by two-tailed Student's *t*-test

bioRxiv preprint doi: https://doi.org/10.1101/2025.09.08.674524; this version posted September 10, 2025. The copyright holder for this preprint (which was not certified by peer review) is the author/funder, who has granted bioRxiv a license to display the preprint in perpetuity. It is made available under aCC-BY 4.0 International license.

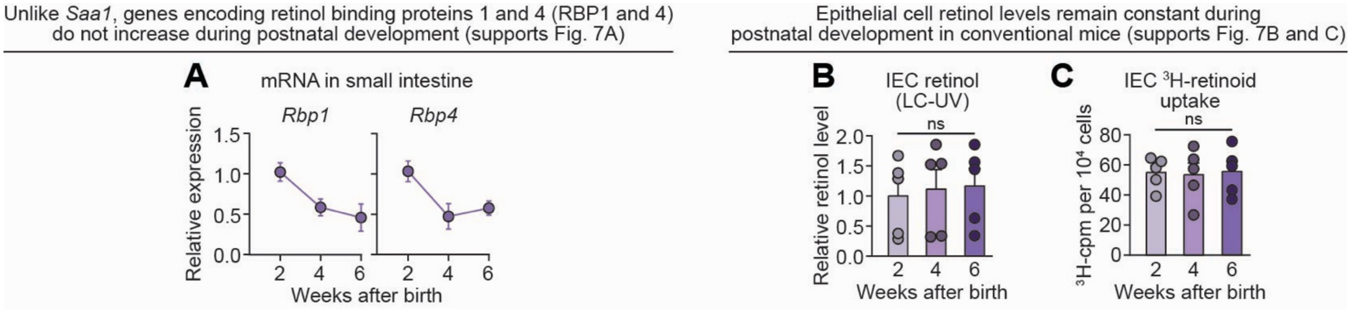

Figure S7: *Rbp* expression and retinoid uptake in intestinal epithelial cells across postnatal intestinal development (supports Figure 7).

(A) qPCR analysis of *Rbp1*, and *Rbp4* mRNA expression in the small intestines of 2-, 4-, and 6-week-old conventional wild-type mice. n = 5 mice per group per timepoint.

(B) Relative retinol content measured by LC-UV analysis of IECs isolated by EDTA treatment from 2-, 4-, and 6-week-old conventional wild-type mice.

(C) ^{3}H-retinoid levels in IECs 6 hours after gavage in 2-, 4-, and 6-week-old conventional wild-type mice.

IEC, intestinal epithelial cells; RBP, retinol-binding protein. n=5 mice per group. Data are representative of two independent experiments. Means ± SEM are plotted. ns, not significant by two-tailed Student's t-test.

bioRxiv preprint doi: https://doi.org/10.1101/2025.09.08.674524; this version posted September 10, 2025. The copyright holder for this preprint (which was not certified by peer review) is the author/funder, who has granted bioRxiv a license to display the preprint in perpetuity. It is made available under aCC-BY 4.0 International license.

Supplementary tables

Table 1

Time (min)	Mobile A (%)	Mobile B (%)
0.0	100	0
2.5	0	100
8.5	0	100
8.6	100	0
10.0	100	0
